# Arboreal Day Geckos (*Phelsuma madagascariensis*) Differentially Modulate Fore- and Hind Limb Kinematics in Response to Changes in Habitat Structure

**DOI:** 10.1371/journal.pone.0153520

**Published:** 2016-05-04

**Authors:** Mingna V. Zhuang, Timothy E. Higham

**Affiliations:** Department of Biology, University of California Riverside, Riverside, California, 92521, United States of America; Brown University, UNITED STATES

## Abstract

By using adhesion, geckos can move through incredibly challenging habitats. However, continually changing terrain may necessitate modulation of the adhesive apparatus in order to maximize its effectiveness over a range of challenges. Behaviorally modulating how the adhesive system is applied can occur by altering the alignment of the foot relative to the long axis of the body and/or the angles between the digits (interdigital angle). Given the directionality of the adhesive system, geckos likely vary the application of the system via these mechanisms as they run. We quantified 3D movements (using high-speed video) of the day gecko, *Phelsuma madagascariensis*, running on a range of ecologically relevant inclines (0°, 45°, 90°) and perch diameters (1.5 cm, 10 cm and broad). We measured the instantaneous sum of interdigital angles and foot alignment relative to the body, as well as other kinematic variables, throughout each stride and across treatments. Modulation of foot alignment at 45° and 90° was similar between the forelimb and hind limb, but differed at 0°, suggesting that *P*. *madagascariensis* is able to exert an adhesive force using multiple strategies. Both the sum of interdigital angles and alignment in the fore- and hind foot were modulated. Differences in modulation between the limbs are likely related to the underlying morphology. The modulation of interdigital angle and foot alignment suggests that aspects other than the mechanism of adhesion, such as joint morphology, are important for arboreal movement in geckos. Our study of foot usage in arboreal locomotion reveals patterns that may be widespread across pad-bearing lizards. In addition to understanding the constraints exerted by the adhesive apparatus, we highlight how biomechanical traits may respond to the evolution of novel adaptations and morphologies.

## Introduction

Patterns of terrestrial locomotion differ considerably among taxonomic, temporal, and spatial scales. Lizards are especially adept at moving on myriad different substrates and have numerous specializations in their feet for doing so. For example, sand-dwelling lizards, such as those from the genus *Uma*, often have toe fringes to maximize surface area [1, 2]; chameleons have the ability to grasp small branches and/or rocky projections [3, 4]; and anoles and geckos often have a dry adhesive system for increasing friction on smooth surfaces [5–8]. The locomotor behavior of lizards on a variety of substrates including sand, water, and arboreal branches/trunks has been examined in several taxa [1, 9–11], and differences among these taxa suggest that lizard locomotion is variable [12]. These dynamic changes in locomotion are constrained by the morphology underlying the locomotor system, especially the foot, which transfers force from the animal to the substrate. Although the variation in kinematics of the more proximal joints has been described, detailed foot kinematics remain relatively understudied.

The foot is the first point of contact with the substrate and is the mechanical unit that transmits force to the ground during locomotion [13]. The foot also maintains locomotor stability [14] and generates propulsive forces [15]. Although these functions have behavioral lability on varying substrates, morphological modifications determine the limits within which behavior can be modulated. The foot is often a site for morphological modification, resulting in deviations from the typical lizard foot [16]. These changes in foot morphology have consequences for locomotion. Thus, understanding how the foot behaves is a necessary component for understanding the link between morphology and biomechanics [17].

Geckos possess one of the most intricate and complex examples of foot modification. The evolution of a unique directional adhesive system in this lineage is accompanied by a number of morphological changes, as well as the evolution of a digital hyperextension system that fundamentally changes how the foot is deployed and disengaged during locomotion [18, 19]. When this active adhesive apparatus is used, the digit tips are the first to disengage with the substrate, instead of being the last. Furthermore, an increase in the interdigital angles and shortening of the digits [20] suggests a morphological departure from the typical lizard foot described by Rewcastle [21], and this difference in morphology may drastically affect locomotion and adhesion. This increase in the overall sum of interdigital angles should allow an increased range of motion of the individual digits in comparison to the typical lizard foot. Given the directionality of the gecko adhesive system [22–25], understanding how the foot is oriented during locomotion and how the adhesive system is applied is important.

How the application of the adhesive system is modulated is a key question that has been poorly addressed. Most geckos have a directional adhesive system, meaning that stronger forces of adhesion are achieved when setae are loaded towards the proximal portions of the foot [22, 24, 25]. This is especially important for geckos that not only must coordinate the application of adhesion between their limbs, but also modulate adhesion in response to habitat structure. Autumn et al. [26] found differences in the time of adhesive system engagement in the forefoot and hind foot during climbing locomotion. Modulation of the adhesive system may also occur in response to changes in incline. Wang et al. [27] examined locomotion on vertical and inverted substrates in Tokay geckos and suggested that modulation of the interdigital angles and foot placement ensures that adequate adhesive force is applied in order to counter the effect of gravity. On vertical substrates, digits II, III and IV generate most of the shear forces in the forefoot while digits I, II and III generate most of the shear forces in the hind foot. During static adhesion, geckos modulate digit position so that some of the adhesive system is always aligned in to counter the effect of gravity [28].

Studies of geckos moving on broad and inclined substrates have revealed the general limb kinematics of gecko locomotion [19, 26, 29, 30]. However, climbing substrates vary in both diameter and incline. How the gecko adhesive system is applied is likely to change given that these complex surfaces constrain how limbs can be placed, and curved surfaces result in elevated medio-lateral forces [31]. Therefore, we examined the application of the gecko adhesive apparatus on complex arboreal substrates in order to understand how habitat structure constrains and facilitates locomotion. We examined both forelimb and hind limb kinematics in response to changes in perch diameter and incline, with a focus on the foot kinematics in a clawless arboreal specialist, *Phelsuma madagascariensis*. We hypothesized that foot and limb kinematics would differ depending on perch diameter due to the altered medio-lateral forces [31]. As a result, geckos should respond to narrower perch diameters by increasing humerus depression and rotation, and decreasing humerus retraction [32]. We also expected increased femur rotation and retraction on narrower perch diameters. Additionally, we hypothesized that geckos would adopt a more sprawled posture on vertical substrates, which would result in decreased femur depression, increased femur protraction and increased ankle extension [28]. Decreased humerus retraction, increased elbow extension, and increased knee flexion were also expected on the vertical substrates.

When stationary on vertical substrates, geckos modulate foot orientation and digit position so that some of the digits directly oppose gravity, ensuring passive loading of the setae [28]. Therefore, with increased inclines, alignment of the foot with the antero-posterior axis should be greater, and the sum of the interdigital angles should decrease in order to increase the number of digits loaded in opposition to gravity.

## Materials and Methods

This study was carried out in strict accordance with the recommendations in the Guide for the Care and Use of Laboratory Animals of the National Institutes of Health. Our protocol was approved by the Institutional Animal Care and Use Committee (IACUC) of the University of California, Riverside (Protocol Number: A-20110038). We obtained five juvenile *Phelsuma madagascariensis* (mass = 17.53g—27g; snout-vent length (SVL) = 6.9 cm—9.3 cm) from commercial suppliers (Gecko Ranch, Durham, NC and Exotic Pets, Las Vegas, NV). This species is either clawless or has vestigial claws and often occupies both broad and narrow surfaces in palm tree environments [33].

### Experimental procedure

**Fig 1:**
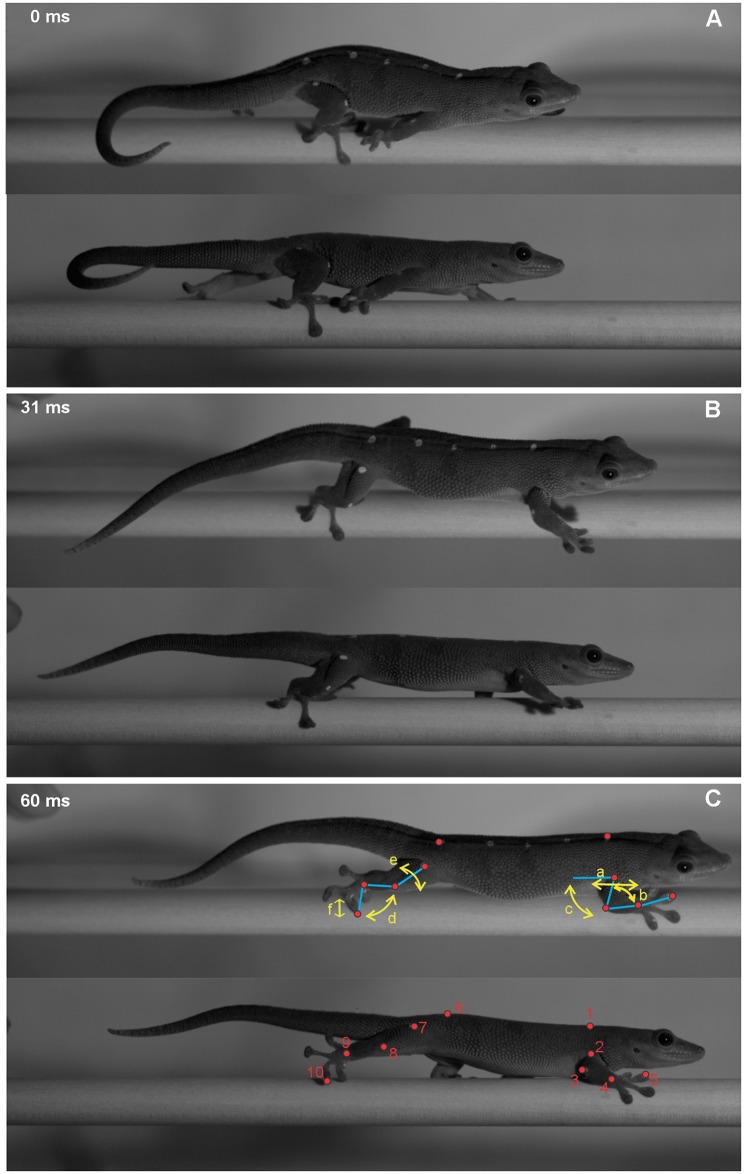
Oblique and lateral views of *Phelsuma madagascariensis* at footfall (A), midstance (B) and endstance (C) on the 1.5 cm perch diameter inclined at 45°. The gecko is moving at approximately 0.71 ms^-1^. Joint angles calculated (yellow arrows) include humerus/femur retraction (a), knee/elbow angle (b), humerus/femur depression (c), wrist/ankle angle (d), humerus/femur rotation (e) and vertical tip position of digit III (f). Landmarks (red dots) used in the calculations of joint angles are as follows: midline of the pectoral girdle (1), shoulder (2), elbow (3), wrist (4) tip of digit III of the forefoot (5), middle of pelvic girdle (6), hip (7), knee (8), ankle (9), and tip of digit III of the the hind foot (10).

Lizards were marked with white nail polish on the dorsal body, shoulder, hip, elbow, knee, wrist and ankle joints to facilitate digitizing kinematic data (Fig 1). Lizards ran on 1.5 cm and 10 cm diameter wooden dowels and a broad wooden trackway made of plywood. Each substrate was inclined at 0°, 45° and 90°. This range of dowels was chosen with respect to a similarly sized species’ ecology [34]. The dowel was suspended 1.1 m above ground by a wooden board that rotated on a wood base. Because *P*. *madagascariensis* can autotomize its skin, precautions to reduce handling were taken by using a black plastic tube with hinged doors on both ends of the tube. The tube was placed at both ends of the setup, with one door open to simulate a dark hiding spot. The lizard was placed in the tube and encouraged to walk onto the top of the dowel with the prod of a thin wooden dowel through the tube. From there, the lizard was encouraged to run by tapping the tail or body lightly. Once the gecko entered the tube at the other end, it was removed and switched with the tube at the other end in order to take additional trials without disturbing the gecko’s skin. After some training, the lizards ran readily across the dowel into the tubes. To prevent lizard escape, the dowel on which the lizard ran was also surrounded by an enclosure of 0.635 cm thick plexiglass. Individuals were run no more than 10 times a day with 2 minutes of rest between trials.

Two high-speed video cameras (Phantom, Wayne, NJ, USA) simultaneously captured the oblique and lateral views at 1600 Hz. This frame rate was required in order to capture adequate digit detail. Video files were downsampled to 800 Hz (Final images per cycle = 111 ± 27 images). For the forelimb and hind limb of each individual, we captured three strides in which the lizard was moving steadily across the top of the perch.

### Kinematics

Digitization of sequences to obtain three-dimensional coordinates of each landmark was performed using DLT DV 3 [35]. In addition to a variety of points on the body and joints, the surface of the perch was digitized to observe where the limbs moved in relation to the perch. The x-axis of each trial indicated the antero-posterior movement, parallel to the direction of locomotion, positive towards the anterior. The y-axis was perpendicular to the x-axis, vertical to the surface and positive dorsal to the lizard. The z-axis described mediolateral movement and was positive into the view.

Three trials were obtained for each treatment per individual. A total of 134 trials were analyzed. Landmark data were obtained for the following: five markers on the dorsum, right shoulder and hip, knee and elbow, ankle and wrist, and the tips of digits 2–5 in the forefoot and hind foot. Because digit I is reduced in *P*. *madagascariensis*, it was not digitized and angles involving digit I were not calculated. A spline of 5x10^-9^ was applied to all landmark data except for the digit tips. A more conservative spline was applied to the digit tip landmarks of 5x10^-10^. Calculations of instantaneous joint and digit angles at footfall, mid stance, and end stance were performed using custom-written code in MATLAB (R2013b, The Mathworks, Natick, MA, USA) [36]. Although we used external landmarks, we assumed that these accurately represented changes in angles between respective bones. For example, depression of the shank will be interpreted as femur depression.

General kinematic variables were calculated using body markers. Speed was calculated using a landmark on the midline of the body (center of the pelvic girdle). The total distance traveled was divided by the duration between frames and was standardized by dividing by SVL. Stride length was the distance traveled on the x-axis in a complete stride cycle, standardized by dividing by SVL. Stride frequency was the number of strides completed per second (Hz). We also digitized the tip of digit III in relation to the top of the substrate, such that a negative number indicates the digit tip is beneath the top of the perch.

**Fig 2:**
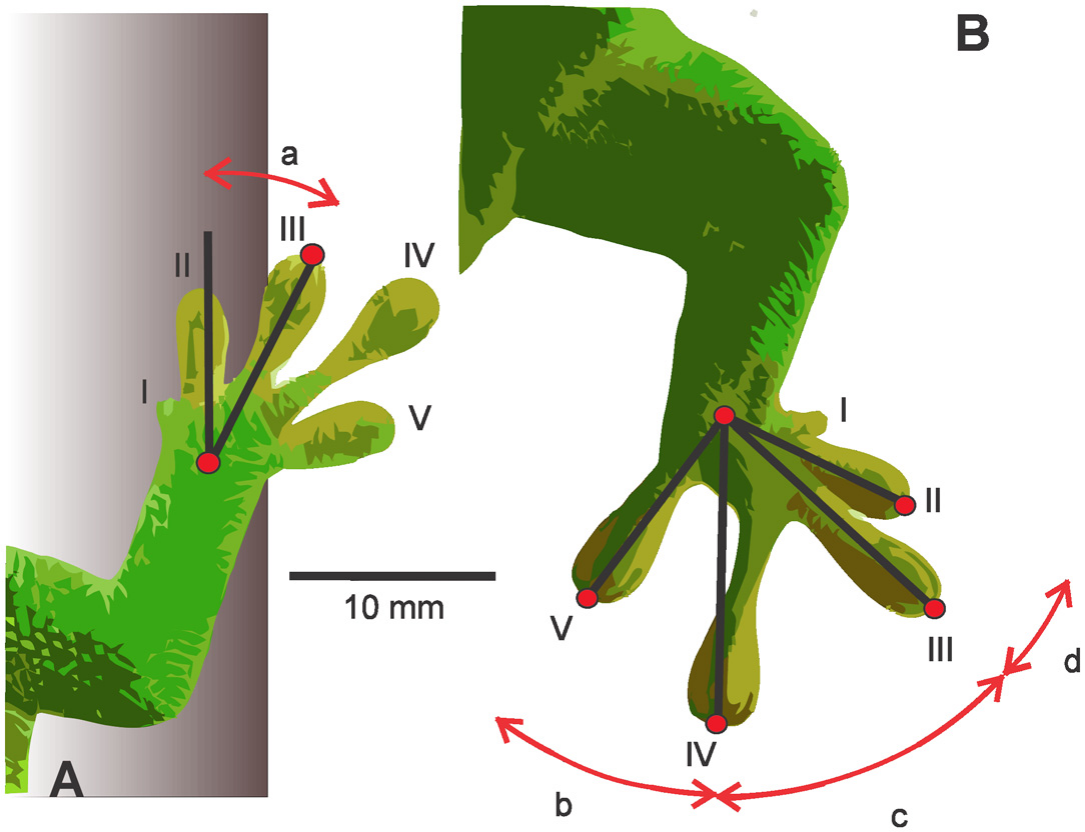
Dorsal images of the forefoot (A) and hind foot (B) of *P*. *madagascariensis*. The scale bar is 10 mm. Foot alignment (a) is calculated as the two-dimensional angle between the axis of the foot and the antero-posterior axis. Interdigital angles (b-d) are calculated as the angle between the line connecting the wrist/ankle joint to the tip of the digit and the line connecting the wrist/ankle joint to the adjacent digit tip.

Three-dimensional joint angles in the hind limb and forelimb (elbow, knee, wrist and ankle) were calculated using previously published methods [32, 37, 38] (Fig 1). Greater flexion is indicated by smaller angles between 0° and 180°. Humerus and femur depression were calculated as the three-dimensional angles between the horizontal plane containing the shoulder/hip joint and the humerus/femur. Positive angles indicate increased depression. Foot depression was the three-dimensional angle between the horizontal plane containing the wrist/ankle joint and the axis of the foot. Humerus and femur retraction was calculated as the two-dimensional angle between the humerus/femur and the line running from the body marker placed between the pectoral/pelvic girdle and the shoulder/hip joint. Positive angles indicate retraction, where the elbow/knee is posterior to the shoulder/hip. Humerus/femur rotation was calculated as the three-dimensional angle between the vertical plane of the humerus/femur and the plane including the humerus/femur and the radius and ulna/ tibia and fibula. Three-dimensional angles between the digits were calculated by measuring the angle between the axes running from the digit tips through the ankle/wrist marker (Fig 2). The instantaneous sum of interdigital angles (digit II to V) was then calculated, such that smaller values indicate that the digits are oriented in a similar direction. The vertical tip position of digit III was calculated as the lowest point of digit III during the stride from the top of the perch.

Foot alignment was calculated by measuring the angle between the axis of the foot (digitized by the line from the wrist/ankle to the tip of digit III) and the antero-posterior axis running through wrist or ankle, respectively (Fig 2). A larger number indicates that the foot is oriented perpendicular to the body and the alignment with the antero-posterior axis of the body has decreased. Smaller numbers indicate that the foot is positioned more parallel to the antero-posterior axis and therefore, has greater alignment. Negative numbers indicate that the foot is less aligned and inverted. Because of the directional adhesive system and the head-up body position in this study, we interpreted greater alignment as orienting the foot to counteract the effect of gravity.

### Statistical analysis

Statistical analyses were performed in SPSS Version 22, and we used *p*<0.05 as the threshold for statistical significance. Because speed was affected by perch diameter and incline, we regressed variables against body speed, and took the residuals of variables that had statistically significant relationships (*p*<0.1, one tail). We kept the rest of the variables as they were originally calculated. The raw data was lastly averaged across three strides per treatment and individual for use in further analyses.

We first performed a principal components analysis (PCA) to reduce dimensionality on the forelimb and hind limb variables separately, including footfall, mid and end stance variables (Table 1). We selected principal components that had an eigenvalue of greater than 1. For interpretation of the loadings, we selected variables that loaded above 0.5 on each PC axis. To determine the effects of perch diameter and incline on these axes, we performed a three-way ANOVA with post-hoc Tukey HSD tests (p < .05), using PC scores, the first two PCs. For all ANOVAs, we included the effect of the individual as a random factor and incline and perch diameter as fixed factors. Natural log transformations were applied to variables that did not meet the assumption of equal variances. Because we were particularly interested in foot kinematic variables, we conducted separate three-way ANOVAs post-hoc Tukey HSD tests the foot alignment and sum of the interdigital angles.

**Table 1:** Loadings from a principal component (PC) analysis of kinematic variables performed separately on the forelimb and hind limb.

Forelimb				Hind Limb			
Variable	PC1	PC2	PC3	Variable	PC1	PC2	PC3
Stride frequency*	0.28	0.15	0.07	Stride frequency*	-0.26	-0.05	**0.53**
Stride length*	-0.19	0.47	-0.05	Stride length*	0.13	0.14	-0.01
Humerus depression (ff)	**0.87**	0.05	-0.03	Femur depression (ff)*	**-0.57**	-0.07	-0.37
Humerus depression (ms)	**0.91**	0.10	-0.03	Femur depression (ms)	**-0.91**	-0.01	-0.31
Humerus depression (es)*	**0.79**	0.27	-0.29	Femur depression (es)	**-0.80**	0.01	-0.18
Humerus retraction (ff)	-0.07	-0.06	0.17	Femur retraction (ff)	0.07	-0.25	0.15
Humerus retraction (ms)*	0.14	-0.16	-0.08	Femur retraction (ms)*	0.14	-0.05	-0.04
Humerus retraction (es)	0.23	0.10	-0.06	Femur retraction (es)*	-0.33	-0.06	-0.09
Humerus rotation (ff)	**0.79**	-0.31	-0.03	Femur rotation (ff)	**0.89**	0.15	0.03
Humerus rotation (ms)	**0.81**	-0.38	-0.20	Femur rotation (ms)	**0.93**	0.09	0.01
Humerus rotation (es)*	0.41	-0.40	0.19	Femur rotation (es)	**0.89**	-0.15	-0.02
Elbow angle (ff)*	-0.04	**0.72**	0.25	Knee Angle(ff)*	0.09	0.01	0.10
Elbow angle (ms)*	-0.11	**0.72**	0.22	Knee Angle (ms)	0.11	0.13	0.14
Elbow angle (es)*	0.06	**0.83**	-0.16	Knee Angle (es)	-0.21	0.14	-0.09
Wrist Angle (ff)*	-0.05	0.02	**0.76**	Ankle angle (ff)	0.40	0.20	**0.67**
Wrist Angle (ms)*	-0.02	-0.10	**0.90**	Ankle angle (ms)	0.09	-0.08	**0.88**
Wrist angle (es)	-0.14	0.03	0.32	Ankle angle (es)	-0.07	0.10	0.39
IDA digits II and III (ff)	0.21	-0.03	-0.22	IDA digits II and III (ff)	-0.31	0.01	-0.30
IDA digits II and III (ms)	0.11	-0.09	-0.44	IDA digits II and III (ms)	-0.39	0.31	0.18
IDA digits II and III (es)	0.06	-0.14	-0.25	IDA digits II and III (es)*	0.04	**0.94**	-0.03
IDA digits III and IV (ff)	0.47	0.01	-0.21	IDA digits III and IV (ff)*	0.08	**0.96**	-0.05
IDA digits III and IV (ms)*	0.02	0.04	-0.14	IDA digits III and IV (ms)	0.00	0.26	-0.34
IDA digits III and IV (es)	0.35	-0.11	**-0.53**	IDA digits III and IV (es)	-0.14	0.27	**-0.63**
IDA digits IV and V (ff)*	-0.15	0.05	-0.08	IDA digits IV and V (ff)*	0.06	**0.97**	-0.01
IDA digits IV and V (ms)*	-0.05	0.07	-0.02	IDA digits IV and V (ms)	-0.15	0.42	-0.20
IDA digits IV and V (es)	-0.28	0.01	-0.19	IDA digits IV and V (es)	0.18	-0.11	-0.07
Foot alignment (ff)	-0.48	0.48	**0.56**	Foot alignment (ff)	**0.60**	0.06	**0.55**
Foot alignment (ms)	**-0.53**	0.36	**0.55**	Foot alignment (ms)	**0.65**	-0.04	**0.59**
Foot alignment (es)*	0.14	-0.16	0.12	Foot alignment (es)*	**-0.51**	-0.12	-0.22
Digit III vertical tip distance †*	**-0.69**	**0.54**	-0.23	Digit III vertical tip distance†*	**0.73**	0.01	-0.15

Loadings with a magnitude ≥ .5 are in bold

ff, footfall; ms, midstance; es, endstance

* Variable affected by speed

^†^ Digit III vertical tip position is measured from the lowest position of the digit tip to the top of the substrate

## Results

### Effects of perch diameter and incline on *P*. *madagascariensis*

*Phelsuma madagascariensis* decreased speed on the 1.5 cm perch in comparison to the 10 cm and broad perches (*F*_2,8_ = 24.79, *p*< 0.001) and decreased speed on the 90° treatment in comparison to 0° and 45° (*F*_2,8_ = 10.19, *p*<0.001). The effect of perch diameter did not depend on incline (*F*_4,16_ = 2.38, *p* = 0.07). In the hind limb, the duty factor averaged 0.46±0.10, 0.51±0.06, 0.52±0.09 for 0°, 45° and 90°, respectively across all perch diameters, and averaged 0.55 ± 0.08, 0.49 ± 0.09, 0.45 ± 0.07 for the 1.5 and 10 cm and the broad perches, respectively across all inclines. Duty factor in the forelimb averaged 0.47 ± 0.09, 0.44 ± 0.07, 0.49 ± 0.11 for 0°, 45° and 90° respectively across all perch diameters and averaged 0.52±0.08, 0.44±0.09, 0.43±0.09 on the 1.5, 10 cm and broad perches.

The first three components of the hind foot PCA explained 54.22% (PC1:25.48%, PC2:15.29%, PC3:13.46%). Higher values on PC1 corresponded with greater long-axis clockwise femur rotation, less femur depression at footfall, midstance and endstance, a lower vertical tip position of digit III and decreased foot alignment at footfall and midstance but increased foot alignment at endstance (Table 1). Higher values on PC2 corresponded with greater angles between digits II and III at end stance, digits III and IV at footfall, and IV and V at footfall. Higher values on PC3 corresponded with decreased hind foot alignment at footfall and mid stance, greater ankle joint angles at footfall and midstance, a decreased angle between digits III and IV at end stance and greater stride frequency. Therefore, PC3 represented generally the modulation of more distal hind limb elements.

**Fig 3:**
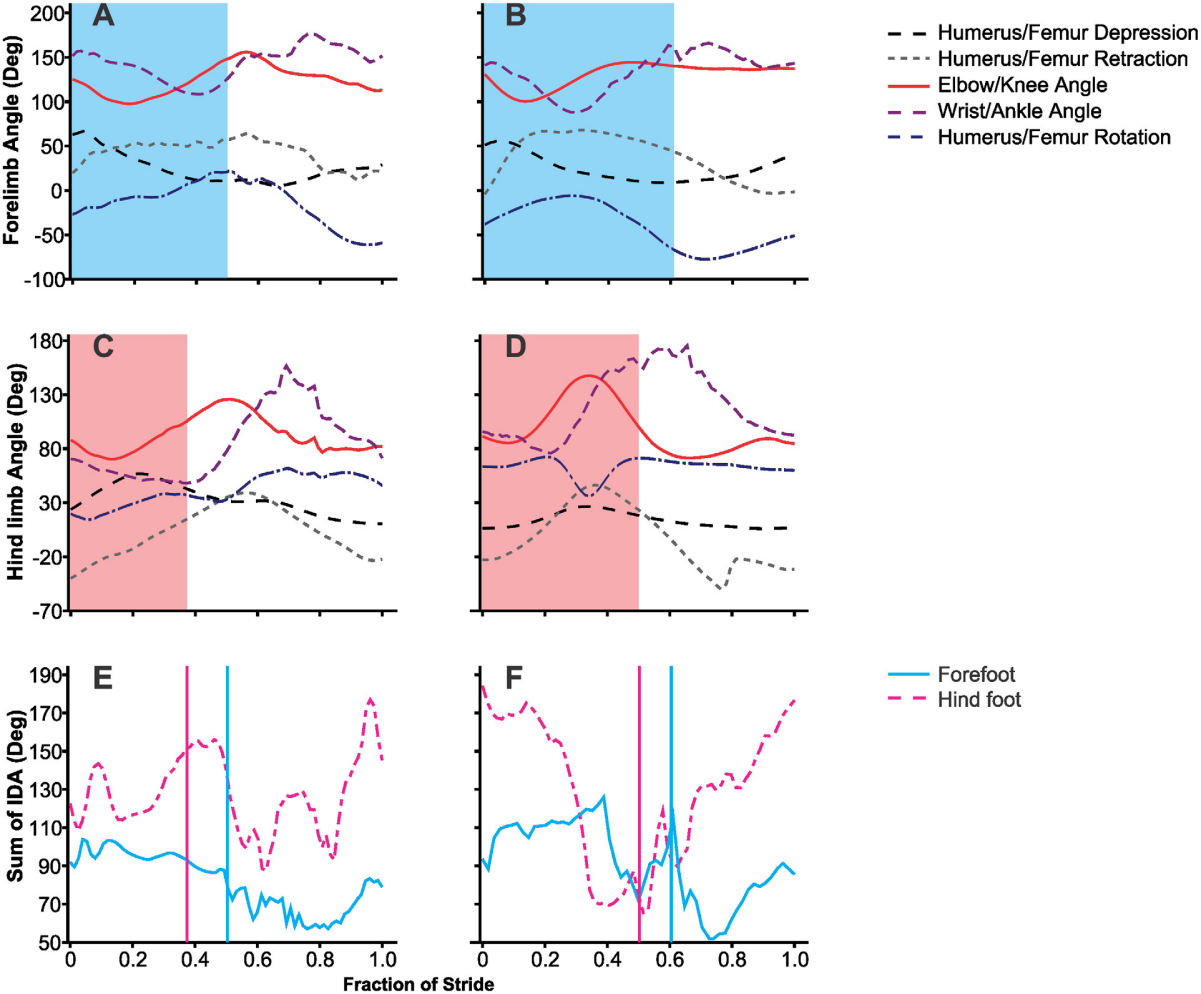
Representative joint angles for the forelimb (A and B) and hind limb (C and D) and sums of interdigital angles (IDA) for the small perch diameter inclined at 90° (A, C and E) (speed = 0.71 ms^-1^) and the broad perch at 0° (B, D And F) (speed = 1.13 ms-1) of the same individual. The shaded regions (A-D) indicate the stance phase and solid vertical lines (E, F) indicate the end of stance phase for the forelimb (blue) and hind limb (pink). The x-axis represents the fraction of the stride. For joint angles, smaller values along the y-axis indicate greater flexion.

**Fig 4:**
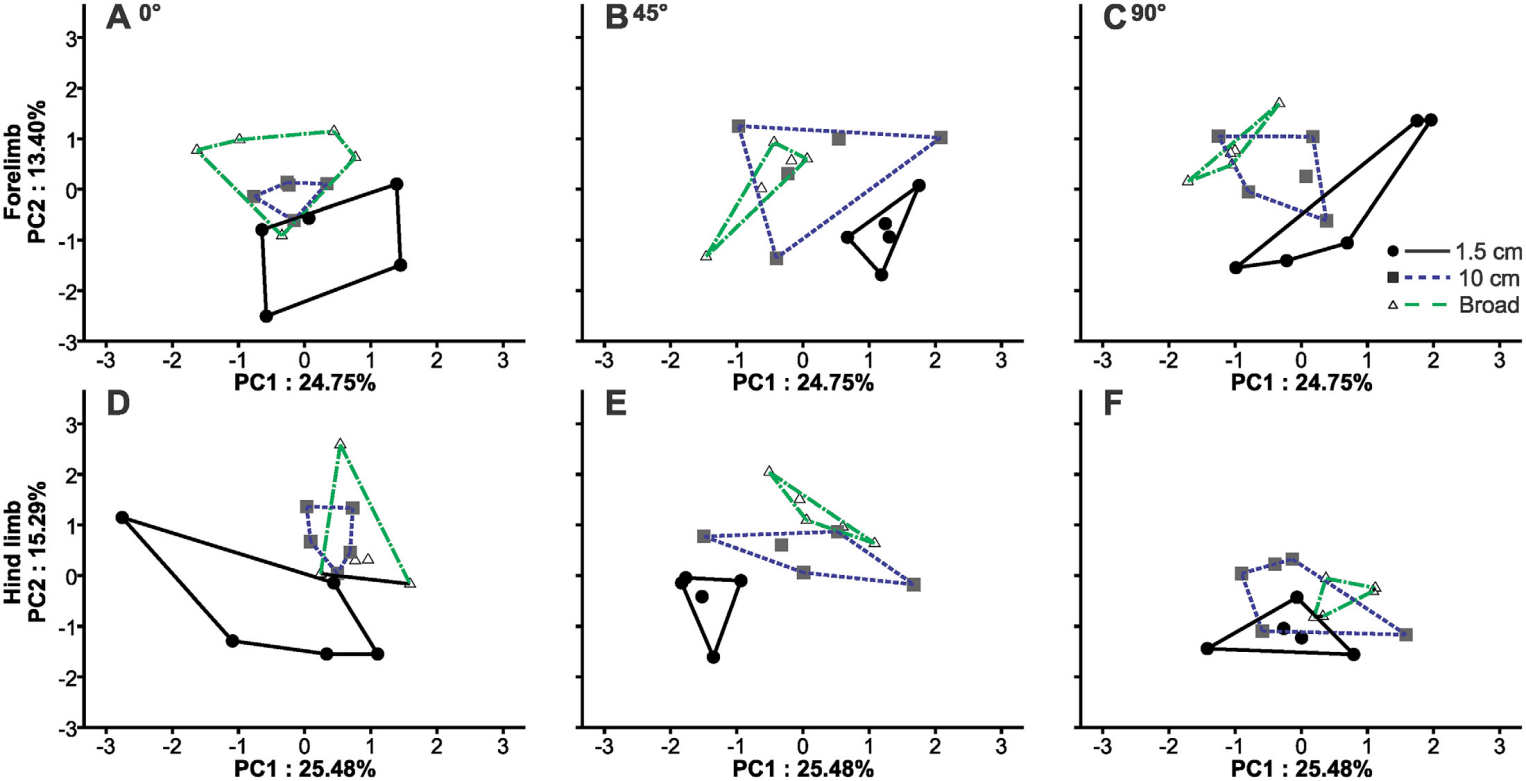
Principal component analysis (PCA) results for the kinematics of the forelimb (A-C) and hind limb (D-F). The first two principal components are plotted with the percent of variance explained by each component. Separate plots are presented for the 0° (A and D), 45° (B and E) and 90° (C and F) treatments. Each point represents the mean value of an individual per condition. For the hind limb, femur rotation and depression loaded strongly on PC1. On PC2, foot alignment and ankle flexion loaded strongly. For the forefoot, humerus depression, vertical tip position of digit III and foot alignment loaded strongly on PC1. Foot alignment and wrist extension loaded strongly on PC2.

**Fig 5:**
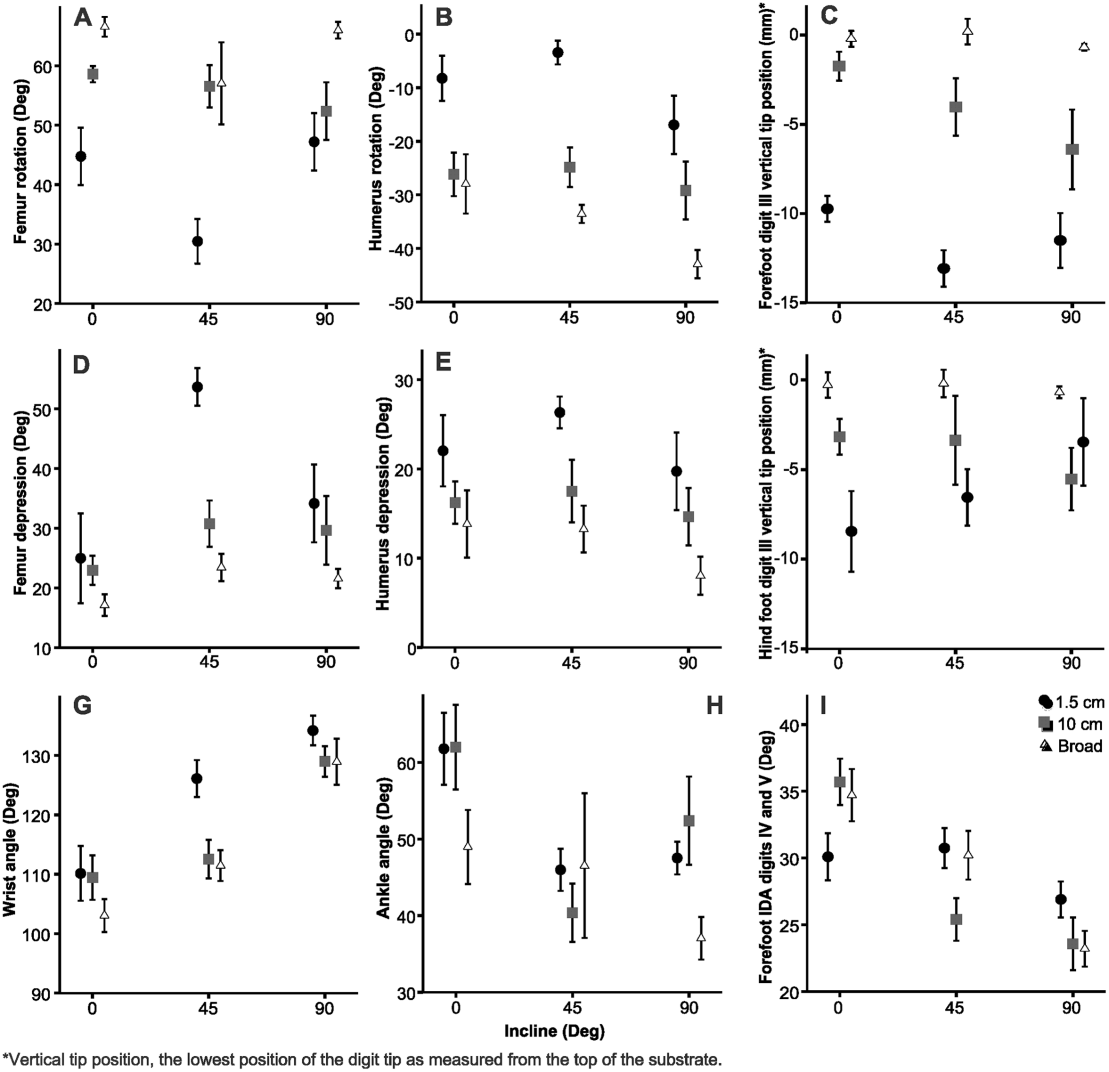
Mean values for several variables that loaded strongly in the principal component analyses for the forelimb and hind limb at mid stance. These include humerus and femur depression, humerus and femur rotation, wrist and ankle angle, angle (IDA) between digits IV and V in the forelimb and the vertical digit tip distance of digit III. Error bars indicate SEM.

**Fig 6:**
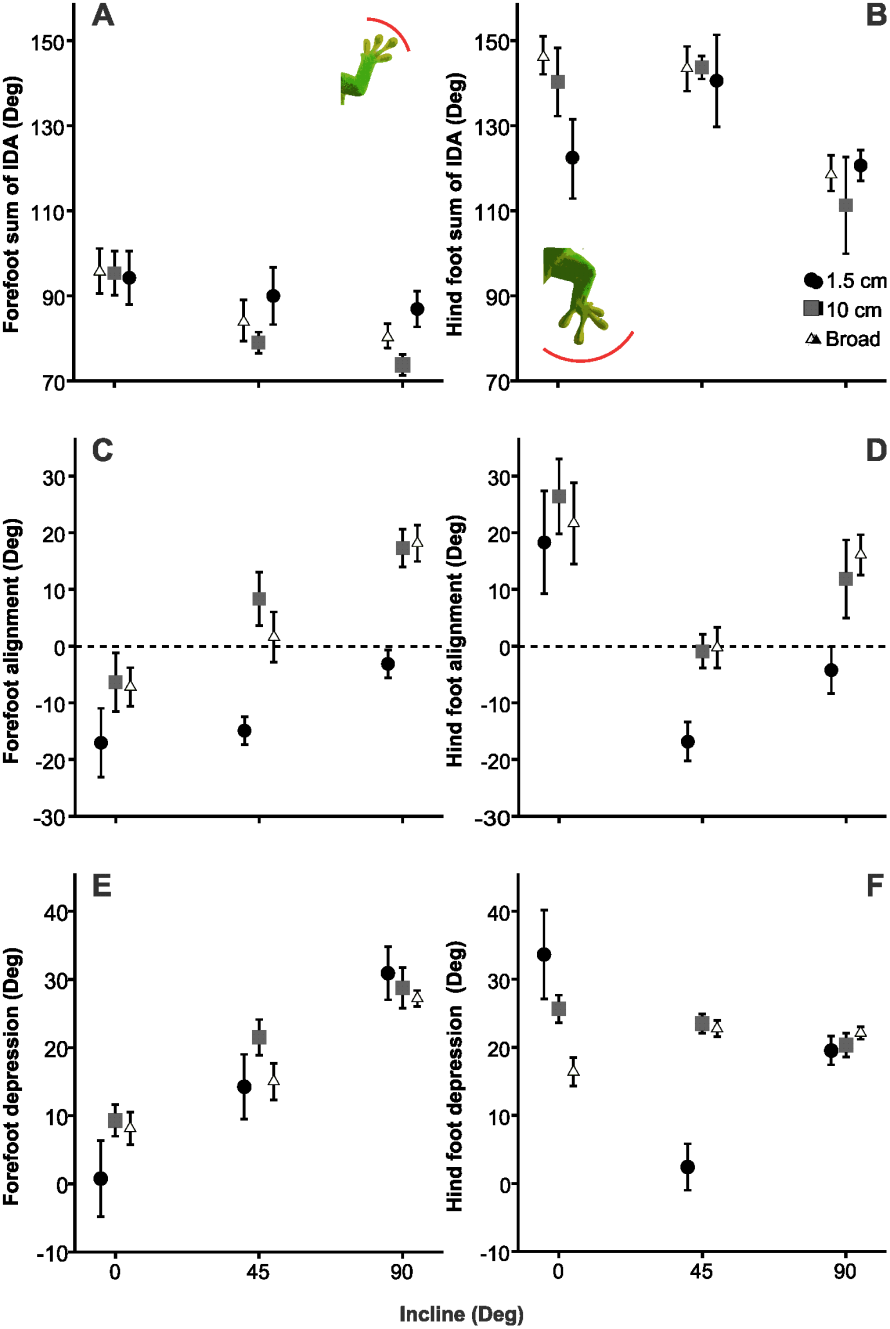
Mean value plots for the sum of interdigital angles (A and B) foot alignment (C and D) and foot depression (E and F) at midstance. Results are shown for the forefoot (A, C and E) and hind foot (B, D and F). Note that decreased values of foot alignment with the z-axis indicate increased alignment with the long-axis of the body. Negative values indicate that the foot is inverted and oriented medially. See methods for a detailed explanation. Error bars indicate SEM.

Geckos exhibited greater femur depression and less long-axis femur rotation (PC1) on the 1.5 cm perch than the 10 cm and broad perch (*F*_2,8_ = 9.14, *p* < .001; Figs 3–5). Femur motion (PC1) was not significantly affected by incline (*F*_2,8_ = 2.75, *p* = 0.08). The interaction term was not statistically significant (*F*_4,16_ = 1.03, *p* = 0.41). Modulation of the interdigital angles (PC2) was affected by incline (*F*_2,8_ = 11.69, *p*<0.001; Fig 4) and perch diameter (*F*_2,8_ = 16.41, *p*<0.001). The interaction term was not statistically significant (*F*_4,16_ = 1.43, *p* = 0.25). Interdigital angles (PC2) were greater on the 10 cm and broad perch than that on the 1.5 cm perch and were greater at the 0° and 45° treatments than that on the 90°. PC2 varied among individuals (*F*_4,32_ = 3.78, *p* = 0.01). Foot alignment, ankle angles and stride frequency (PC3) were greater on the 0° than at the 45° and 90° treatments (*F*_2,8_ = 7.18, *p* = 0.003; Figs 4–6). PC3 was not significantly affected by perch diameter or the interaction term (*F*_2,8_ = 0.76, *p* = 0.48, *F*_2,8_ = 1.62, *p* = 0.19; Fig 4).

For the forefoot PCA, the first three components explained 48.50% of the variation in kinematics (PC1: 24.75%, PC2:13.39%, PC3:10.35%). Higher values on PC1 corresponded with greater humerus depression at footfall, mid and end stance, long-axis clockwise humerus rotation at footfall and mid stance, increased foot alignment at midstance, and a lower vertical tip position of digit III (Table 1). Higher values on PC2 corresponded with greater elbow angles and a lower vertical tip position of digit III. Higher values on PC3 corresponded with greater wrist angles at footfall and mid stance, a smaller angle between digit III and IV at end stance and decreased foot alignment at footfall and mid stance.

Humerus depression, rotation and foot alignment (PC1) were increased with decreasing perch diameter (*F*_2,8_ = 12.19, *p*<0.001; Fig 4). Humerus motion and foot alignment (PC1) was not significantly affected by incline (*F*_2,8_ = 2.07, *p* = 0.14). The interaction term was not statistically significant (*F*_4,16_ = .66, *p* = 0.62; Fig 5). PC1 varied among individuals (*F*_4,32_ = 3.58, *p* = 0.02). Elbow angle (PC2) was greater on the 10 cm and broad perch than that on the 1.5 cm perch (*F*_2,8_ = 2.07, *p* = 0.14). Modulation of the elbow joint was not affected by incline or the interaction term, but did vary among individuals (*F*_2,8_ = 1.90, *p* = 0.17, *F*_4,16_ = .77, *p* = 0.56, *F*_4,32_ = 9.26, *p* < .001). Foot alignment decreased and wrist angle (PC3) increased with increasing inclines (*F*_2,8_ = 25.98, *p*<0.001). Modulation of the distal elements was not affected by perch diameter *(F*_2,8_ = 0.97, *p* = 0.39). The interaction term was not statistically significant (*F*_4,16_ = 0.37 *p* = 0.83).

### Foot kinematics in response to perch diameter and incline

The sum of interdigital angles in the hind foot remained approximately the same throughout stance until the onset of the swing phase, at which time digital spread decreased (Fig 3). This sum decreased in the forefoot after mid stance. The instantaneous sum of interdigital angles in the hind foot was usually greater than the forefoot throughout the stride and on average, the sum of interdigital angles in the hind foot was greater than that of the forefoot *(F*_1,4_ = 248.42, *p*<0.001; Fig 6). Additionally, the effect of incline depended on the limb (*F*_2,8_ = 5.25, *p*<0.001). In the hind foot, the sum of interdigital angles was larger at 45° than the 0° and 90°. On the other hand, the sum of interdigital angles in the forefoot decreased with an increase in incline (Fig 6). In the forefoot, the angle between digits II and III had the highest coefficient of variation when compared to other interdigital angles across most treatments except on the 10 cm perch at 45° and 90° (Table 2). On these latter treatments, the angle between digits III and IV had the greatest amount of variation. In the hind foot, which interdigital angle had the most variation depended on the treatment.

**Table 2:** Averages and coefficients of variation (CV) of interdigital angles in the forelimb and hind limb in response to 1.5 cm, 10 cm and broad perches at 0°, 45° and 90°.

Limb	Interdigital Angle	Incline (Deg)	1.5 cm		10 cm		Broad	
			Mean	CV	Mean	CV	Mean	CV
		0	30.09	0.22	35.71	0.18	34.70	**0.21**
	Angle II-III	45	30.75	0.18	25.40	0.24	30.20	0.23
		90	26.90	0.18	23.57	**0.31**	23.20	0.22
		0	36.78	0.31	26.47	0.27	23.07	0.20
Forelimb	Angle III-IV	45	36.39	0.17	23.99	**0.31**	21.01	0.26
		90	31.47	0.16	20.05	0.19	25.55	**0.28**
		0	27.37	**0.34**	33.17	**0.28**	38.05	0.20
	Angle IV-V	45	22.84	**0.42**	29.60	0.19	33.00	**0.28**
		90	28.38	**0.19**	30.17	0.26	31.85	0.22
	Angle II-III	0	31.93	**0.43**	30.27	**0.25**	31.45	0.21
		45	28.70	***0.33***	29.84	**0.30**	25.33	**0.34**
		90	25.51	0.24	22.59	0.25	18.64	**0.40**
	Angle III-IV	0	41.60	0.22	56.76	0.20	57.67	**0.28**
Hind limb		45	59.10	0.18	52.94	0.22	50.94	0.33
		90	49.28	0.17	30.90	**0.38**	39.17	0.28
		0	48.69	0.35	53.26	0.20	57.47	0.25
	Angle IV-V	45	52.80	0.30	60.94	0.21	67.14	0.21
		90	46.35	**0.28**	57.81	0.30	61.04	0.20

The highest coefficient of variation per treatment is in bold

Foot alignment did not differ between the broad and 10 cm perch, but it was significantly less aligned in both of these treatments compared to the 1.5 cm (*F*_2,8_ = 13.15, *p*<0.001; Fig 6) Foot alignment did not differ between the 0° and 45° treatments, but it was significantly greater in both of these treatments compared to 90° (*F*_2,8_ = 11.56, *p*<0.001). The interaction term was not statistically significant (*F*_4,16_ = 0.604, *p* = 0.662). The foot was directed towards the midline of the body (foot alignment was < 0°) on the 1.5 cm perch at 0°, 45° and 90° and all perches at 0° (Fig 6). Hind foot alignment was greater on the 45° treatment relative to the 0° and 90° treatments (*F*_2,8_ = 11.30, *p*<0.001; Fig 6). The feet were less aligned on the 1.5 cm perch compared to the 10 cm and broad perches (*F*_2,8_ = 4.12, *p* = 0.025; Fig 6). The interaction term was not statistically significant (*F*_4,16_ = 0.43, *p* = 0.77). Foot alignment increased or the foot increased inversion with increased humerus/femur depression (*r* = -0.49, *p*<0.001, *r* = -0.77, *p*<0.001, respectively).

Forefoot depression increased with increasing incline (*F*_2,8_ = 30.51, *p*<0.001), but was not affected by perch diameter (*F*_2,8_ = 1.21, *p* = 0.31). The interaction term was not statistically significant (*F*_4,16_ = 0.43, *p* = 0.77). Hind foot depression was affected by perch diameter and incline (*F*_2,8_ = 5.90, p = 0.007, *F*_2,8_ = 8.06, p = 0.001, respectively). The effect of perch diameter on hind foot depression depended on incline (*F*_4,16_ = 17.05, *p*<0.001). On the 0° treatment, foot depression increased with decreasing perch diameter. On the 45° treatment, foot depression was greater on the broad and 10 cm perch than that on the 1.5 cm (Fig 6).

The sum of the interdigital angles differed among individuals for the forefoot, but not the hind foot (*F*_4,32_ = 3.27, *p* = 0.02, *F*_4,32_ = 1.28, *p* = 0.30, respectively). The sum of forefoot interdigital angles was less on the 45° and 90° treatments than that on the 0° treatment (*F*_2,8_ = 10.10, *p*<0.001; Fig 6). However, the sum was not affected by perch diameter and the interaction term was not statistically significant (*F*_2,8_ = 2.75, *p* = 0.08, *F*_4,16_ = .98, *p* = 0.43, respectively) The sum of interdigital angles in the hind foot was greater on the 0° and 45° treatments than that at 90° (*F*_2,8_ = 4.96, *p* = 0.01). Neither perch diameter, nor the interaction term affected the sum of interdigital angles in the hind foot (*F*_2,8_ = 0.34, *p* = 0.71, *F*_4,16_ = .83, *p* = 0.52, respectively; Fig 6). In both limbs, the sum of interdigital angles decreased as alignment decreased (*r* = -.654, *p* < .001 *r* = -0.33, *p* = 0.029, respectively).

## Discussion

The directional adhesive system of geckos is an innovation that permits the exploitation of smooth vertical surfaces. Geckos can overcome these climbing challenges by loading the adhesive system passively with the digits oriented in opposition to the force of gravity and/or actively by pulling the digits towards the midline of the body [26, 28]. Both of these require that the feet and digits be oriented to maximize the utility of the apparatus [6, 23, 39]. In our study, *Phelsuma madagascariensis* modulated the positions and motions of the forefoot and hind foot in response to changes in perch diameter and incline by altering foot alignment and digital spread. The modulation of foot alignment in both limbs was similar on more inclined surfaces. However, at 0°, the forefoot was inverted and the hind foot was everted. Differences in hind foot and forefoot kinematics suggest differences in contributions to stability and adhesion during locomotion at 0°, which may be related to digital configurations of the forefoot and hind foot, as well as constraints imposed by more proximal elements of the limb (Fig 2).

Russell and Oetelaar [28] observed the limb and digital modulation of *Chondrodactylus bibronii* during stationary adhesion in several orientations (head-up, head-down, laterally facing to the left, and laterally facing to the right). By measuring interdigital angles and the alignment with the digits to the vector of gravity, they found that modulation of digit orientation during stationary adhesion on a vertical substrate allows the passive loading of at least several digits. The large resting sum of interdigital angles (almost 180°) within the limbs of *C*. *bibronii* likely facilitates adhesive application in any body orientation via increasing the potential for passive loading. However, variation in the digital arrangement among gecko lineages potentially has different kinematic consequences [20]. Our study supports the conclusions about the benefits of a wide digital spread, but also suggests potential benefits to narrower, albeit symmetrical, digital spreads.

### Foot kinematics in response to perch diameter and incline

When moving uphill, animals can only move forward by overcoming the counteracting force of gravity. This is achieved, to some extent, by increasing the frictional forces between the animal and the substrate [40]. A single digit could support the weight of *Phelsuma madagascariensis* on glass, suggesting that, like Tokay geckos, the adhesive system is “overbuilt” [28, 41]. However, engaging more digits to the substrate may be necessary on rougher substrates and/or during dynamic motion due to the limited area of contact [28]. Geckos, which have a friction-based adhesive system, are expected to arrange their digits in direct opposition to gravity via modulation of the digital spread and foot alignment with increasing demands on adhesion [28]. In our study, forefoot and hind foot alignment responded to perch diameter and incline similarly, except for the 0° treatment.

We initially predicted that, with increasing incline, foot alignment should increase and the sum of interdigital angles should be small in order to increase the number of digits effectively oriented in opposition to gravity. The first part of the prediction was only upheld on the broad perch diameters at the 45° incline when compared to the 0° treatment for the hind foot and forefoot (Fig 6). This finding suggests that passive loading via gravity was used for attachment on these treatments and there was a greater reliance on digit III. Although increased alignment appeared to occur on the 90° incline at the 1.5 cm perch, greater foot depression on this treatment indicated that the foot was oriented away from the body and likely wrapped around the perch (Fig 6). As a result, the foot was positioned so that the digits can be engaged via pulling the foot towards the midline of the body. If not loaded by gravity (i.e. oriented more parallel to the antero-posterior axis), the adhesive apparatus of geckos may be loaded by pulling the feet towards the midline of the body when the tips of the digits are more abducted than the proximal portions of the digits. For example, *Hemidactylus garnotii* pulls its limbs toward the midline during vertical climbing [26]. This behavior likely contributes to both propulsion and adhesion [26]. This alternative way of employing the adhesive apparatus likely helps stabilize the animal during locomotion, in addition to increasing the effectiveness of adhesive system application. Although Autumn, Hsieh [26] suggests that this strategy of adhesive engagement should occur on inclined surfaces in order to generate greater forces to engage the adhesive system, it is likely that the demands due to gravity are lower at 45° than at 90°. Therefore, loading the adhesive system by orienting the digits in opposition to gravity was sufficient to engage the adhesive system at 45°. On the 1.5 cm perch at 0°, the forefoot was inverted and the hind foot was everted during locomotion (Fig 6). For this treatment, inversion of the forefoot allows gravity to facilitate the attachment of the adhesive system. On the other hand, eversion of the hind foot positions the foot to be engaged via pulling the foot towards the midline. Inversion of the forefoot is likely facilitated by the relatively upright posture of the forelimb in comparison to the sprawled posture of the hind limb. Given the challenges of locomotion on narrow perches, these results suggest that both limbs can counteract elevated mediolateral forces associated with small perch diameters and contribute to stability during locomotion by employing different strategies. Our findings also suggest that greater reliance on digit II in the hind foot occurs on smaller perch diameters, given that digit II was better positioned to be inverted than digit III due to the large morphological interdigital angles of the hind foot (Fig 2). This differentiation in foot alignment modulation indicated multiple strategies for employing adhesion to alter stability and propulsion in the forefoot and hind foot.

We expected decreased foot alignment on the smallest perch diameters in order to allow some digits to counteract the effect of the medio-lateral force experienced when the foot is not placed on the top of the perch [31]. This prediction was supported in both the forefoot and hind foot (Fig 6). The foot was rarely aligned with the antero-posterior axis. The forefoot was inverted on the small perch diameter at 0° and 45°. Inversion of the hind foot also occurred on the 1.5 cm perch at the 45° incline. On the 1.5 cm at 90°, decreased space to place the limb due to the narrow perch, resulted in greater humerus/femur depression, increased height of the center of mass and therefore less stability on this treatment than that on broader perches. On this treatment, the foot was positioned along the long axis of the body, but oriented perpendicular to the long axis of the body, allowing the gecko to counteract toppling forces in addition to gripping the substrate [26, 42] Likewise, the orientation of the foot on broader perches may have been driven, in part, by more proximal elements. As a result of the sprawled posture that decreased the distance between the center of mass and the substrate, decreased humerus/femur depression on these broader perches in comparison to that on the 1.5 cm perch at 90° oriented the foot away from the body [21].

Meldrum [43] qualitatively observed that, arboreal cercopithecine species (*Cercopithecus pogonias*, *C*. *nictitans* and *Lophecebus albigena*), orient the foot so that digits IV and V are positioned perpendicular to the perch in order to facilitate grasping. On broad and level substrates, the foot aligns with the antero-posterior axis. In these arboreal primates, foot modulation facilitates grasping to counteract mediolateral forces when traveling on small perch diameters and facilitates propulsion for forward locomotion on broad perches. We observed a similar strategy in *P*. *madagascariensis*, but we also observed the geckos employing an inverted foot posture as an alternate strategy.

Based on anatomical studies of the Tokay geckos, *Gekko gecko*, the forefoot of geckos possesses a unique tendon pattern that includes a reduced flexor plate and absence of a sesamoid. This morphology allows a greater capability for grasping than lizards such as *Pogona vitticeps*, which possess a common pattern of tendinous connections that has one or two embedded sesamoids [44]. This morphology may facilitate the deployment of the adhesive apparatus and may facilitate its role in maintaining stability while locomoting on smaller perch diameters via a combination of grasping and adhesion. In *P*. *madagascariensis*, grasping in the hind foot was observed during stationary holding on small perch diameters or when falling off the perch. However, Digits IV and V of the hind foot, which were more likely capable of grasping the perch during locomotion, often remained hyperextended on the 1.5 cm perch. This indicates that *P*. *madagascariensis* relies less on grasping for increased lateral stability during forward locomotion. An examination of tendon morphology is needed to examine the differences in grasping ability of the forelimb and hind limb.

Although foot kinematics are often neglected in studies of locomotor biomechanics, a few studies in several species of terrestrial lizard have examined the orientation of the foot relative to the antero-posterior axis [11, 45, 46]. *Dipsosaurus dorsalis* increases foot alignment so that the fourth digit is aligned with forward locomotion at higher running speeds [45]. Several other species (*Callisaurus draconoides*, *Cnemidophors tigris* and *Phrynosoma platyrhinos*) orient their hind feet approximately 10° away from the plane of forward locomotion at footfall, [46]. This increase in foot alignment allows a greater proportion of the forces produced by plantarflexion to contribute to forward propulsion [45]. However, *Uma scoparia* decreases its foot alignment by approximately 10° more than the other species examined, suggesting inter-specific differences. *Sceloporus clarkii* also does not change its hind foot orientation with changes in speed, although maximum speed was not elicited [11]. Russell and Bels [12] proposed that the inter-specific differences in the modulation of foot orientation may be the result of differences in the anatomy of the mesotarsal joint. Neither forefoot (*r* = -0.39, *p* = 0.65) nor hind foot alignment (*r* = -0.11, *p* = 0.22) was affected by speed in *P*. *madagascariensis*, but was affected by perch diameter and incline. However, a full range of speeds may not have been observed. Our results suggest that modulation of foot orientation is not only beneficial for enhancing propulsion, but also for modulating the application of adhesion in geckos. Modulation of foot orientation should be facilitated in geckos due to the modified astragalocalcaneum, which is suggested to have a greater range of motion about the mesotarsal joint than that of the typical lizard [18]. An examination of mesotarsal and mesocarpal joints in geckos would reveal how they have evolved in concert with the evolution of the adhesive system.

### Consequences of differences in the sum of interdigital angles

The sum of the interdigital angles differentiates between situations where the digits are oriented in multiple directions and those in which the digits are in the same direction. We take this as a proxy of how much of the adhesive system is recruited in the direction of foot orientation. Although the absolute distance between digit II and digit V may be small when wrapped around a small perch, the digits are not oriented in the same direction. Therefore, a large sum of interdigital angles on broader perches will be interpreted similarly to that of a large value on a small perch.

Although increases in the sum of interdigital angles may potentially be attributed to the modulation of a single interdigital angle, we found that the same interdigital angle was not always responsible for the variation in the sum of interdigital angles (Table 2). However, modulation of interdigital angles differed between the forefoot and hind foot. In the forefoot, the angle between digits II and III was the most variable across treatments except for the 10 cm perch at 45° and 90°. For these treatments, the angle between digits III and IV was the most variable. For the hind foot, the angle between digits II and III was most variable on the 10 cm perch across all inclines. The angle between digits III and IV was most variable on the 1.5 cm at 45° 90° and the broad perch at 0°. The angle between digits IV and V was most variable on the 1.5 cm perch, at 0° and broad perch, at 45°. Given the greater resting sum of interdigital angles in the hind foot (Fig 2), modulation of the sum of interdigital angles may be attributed to anatomical differences between feet.

We predicted that more digits would align in a similar direction when the geckos ran on inclines greater than 0° and broader perch diameters (10 cm and broad). This prediction was upheld with regards to incline in the forefoot and the hind foot. At 0°, digits in the forefoot were not recruited in a similar direction. Although a large interdigital sum of angles on the broader perches at 0° may not be relevant to adhesion because digital hyperextension occurs at 0° [19], the large sum of interdigital angles on the 1.5 cm perch is important given that geckos must both propel forwards in addition to counteracting the lateral pull on smaller perch diameters. On this perch treatment, the forefoot and hind foot was inverted, some digits were likely dedicated to counteracting the effect of the medio-lateral forces experienced and some digits were likely dedicated to propulsion. In both feet, the sum of interdigital angles decreased with increasing inclines as a result of gravity becoming opposed to the direction of forward locomotion, which then elevated the demand on the gecko in terms of adhesion. Significant decreases in the sum of interdigital angles with increases in incline occurred at 45° in the forefoot and 90° in the hind foot. Thus, shallower angles led to the digits of the forelimb, and therefore the adhesive system, becoming aligned with gravity, whereas it took steeper angles before the same was true for the hind limb.

Modulation of forefoot and hind foot motion involved both changes in the foot alignment and the instantaneous sum of interdigital angles. The differences in the patterns of modulation between limbs may reflect morphological differences. Although not studied in *P*. *madagascariensis*, Russell [18] described the myological and osteological differences between the forelimb and the hind limb in *Gekko gecko*. Unlike *G*. *gecko*, which has a similar resting sum of interdigital angles in the forelimb and hind limb, *P*. *madagascariensis* possesses a smaller resting sum of interdigital angles in the forelimb than that in the hind limb (Fig 2). As a result, the sum of interdigital angles at midstance in the hind foot almost always greater than that observed in the forefoot. The sum of interdigital angles in the hind foot ranged from approximately 100° to 150°, whereas the sum of interdigital angles in the forefoot was restricted to a smaller range of approximately 70°-100° (Fig 6). In general, the sum of interdigital angles was more variable in the hind foot than in the forefoot. These results suggest that a smaller resting sum of interdigital angles is reflective of its instantaneous sum of interdigital angles during locomotion. The forefoot, which has a sum of inter-metatarsal angles of approximately 87°, is limited in its modulation of interdigital angles and direction of adhesive system recruitment. Modulation of adhesion in the forefoot is therefore more reliant on the modulation of foot alignment. The greater resting sum of the interdigital angles in the hind foot, which has a sum of inter-metatarsal angles of approximately 101°, permits the use of a larger range of interdigital angle modulation. However, fewer digits are then recruited in the direction of foot orientation, potentially reducing the contribution of hind foot adhesion to locomotion overall.

In order to facilitate the operation of the adhesive system, interdigital angles in the hind feet of padbearing lineages are thought to be greater than that of padless lineages [20]. However, studies examining the digital configuration in the forelimb of gecko lineages has not been examined, although variation seems to exist. For example, *C*. *bibronii* not only has a nearly 180° sum of interdigital angle configuration in the hind limb, but also in the forelimb [28]. It is evident that *P*. *madagascariensis* does not possess such a digital configuration in the forelimb (Fig 2). Whereas the 180° range in the forelimb and hind limb of *C*. *bibronii* may allow the gecko to adhere regardless of body orientation, *P*. *madagascariensis* may require more modulation to maintain adhesive system engagement in the same body orientations or may favor some body orientations over others. This might also reflect an arboreal habitat as compared to a rocky habitat that includes more flat surfaces.

### Forelimb and hind limb kinematics of arboreal locomotion

Narrower perches reduce the amount of space on which the foot can be placed and, as a result, the alteration of the proximal limb elements is crucial for accommodating these conditions [37]. Locomotion of *P*. *madagascariensis* was affected more by perch diameter than incline. Furthermore, proximal limb elements appeared to be more important than more distal elements for locomotion, especially in the forelimb (Table 1). These results suggest that there are significant upstream effects of proximal limb elements on more distal limb elements.

In response to decreasing perch diameter, hip and shoulder height decreased in *P*. *madagascariensis*. Decreased hip/shoulder height may have occurred via a number of kinematic changes. Although greater femur and humerus depression occurred on these treatments, the decrease in hip height and shoulder height was likely due to the increased elbow and knee flexion. This response is consistent with previous studies, in which animals responded to smaller perch diameters by lowering the center of mass to increase stability [32, 37, 47–50]. Like *Anolis carolinensis*, geckos increased long-axis humerus rotation and decreased femur rotation with decreasing perch diameter [32] (Fig 5, Table 1). Additionally, greater femur rotation was associated with decreased femur depression. Greater femur rotation may be attributed to the sprawling posture of the hind limb, which requires femur rotation as a mechanism of decreasing rotation necessary at the mesotarsal joint, and during femur retraction, maintaining the knee joint axis perpendicular to the parasagittal plane [21].

Far more studies have examined the effects of incline on locomotor kinematics in lizards than perch diameter [4, 32, 36, 38, 51, 52, 19, 28, 53, 37, 54, 55]. As incline increases, the impact of gravity acts to pull the animal down the slope. Thus, during vertical locomotion, the vertical component of the ground reaction force acts perpendicular to the force of gravity and does not contribute to substrate attachment. The effect of gravity at 90° directly opposes forward locomotion, which causes slipping if there is no increase of friction or engagement of an adhesive system [56]. *Phelsuma madagascariensis* increased wrist extension and decreased forefoot alignment with increasing inclines. Depending on the perch diameter, *P*. *madagascariensis* modulated hind foot alignment and ankle angle. Additionally, unlike *Tarentola mauritanica*, which engages the adhesive system at inclines greater than 10° [19], *P*. *madagascariensis* often did not engage the hind foot adhesive toepads of digits IV and V on steeper inclines and narrower perches. Due to their orientations on these treatments, digits IV and V were not likely to contribute to forward locomotion if engaged and instead, remained hyperextended.

## Conclusion

We explored the function of feet in geckos, an understudied aspect of vertebrate locomotion. *Phelsuma madagascariensis* modulated not only proximal limb elements, but also distal limb elements, in response to changes in perch diameter and incline. Modulation of these elements differed between the forelimbs and hind limbs in ways consistent with observations from previous studies. Furthermore, we identified certain unique behaviors of *P*. *madagascariensis* that may be relevant to its morphology and/or the evolution of the dry adhesive system. Limitations in the range of motion of the individual digits in the forefoot may be related to greater reliance on the modulation of overall foot alignment in comparison to the hind foot. This potential morphological constraint necessitates further examination of foot morphology in concert with kinematic studies examining foot modulation and confirms that digit placement is context dependent during locomotion. Overall, studying foot kinematics in concert with adhesion during locomotion in geckos is critical for revealing potential constraints or ways in which constraints are circumvented. This will help illuminate the evolution of the gekkotan adhesive apparatus.
